# Selenium- and Tellurium-Based Antioxidants for Modulating Inflammation and Effects on Osteoblastic Activity

**DOI:** 10.3390/antiox6010013

**Published:** 2017-02-14

**Authors:** Xi Lu, Gemma Mestres, Vijay Pal Singh, Pedram Effati, Jia-Fei Poon, Lars Engman, Marjam Karlsson Ott

**Affiliations:** 1 Department of Engineering Science, Applied Materials Science, Uppsala University, Box 534, Uppsala 751 21, Sweden; xi.henry.lu@gmail.com (X.L.); pedram.effati@maiiadiagnostics.com (P.E.); 2 Department of Engineering, Microsystems Technology, Uppsala University, Box 534, Uppsala 751 21, Sweden; Gemma.Mestres@angstrom.uu.se; 3 Department of Chemistry, BMC, Uppsala University, Box 576, Uppsala 751 23, Sweden; vijay.singh@kemi.uu.se (V.P.S.); jia_fei.poon@kemi.uu.se (J.-F.P.); Lars.Engman@kemi.uu.se (L.E.)

**Keywords:** antioxidants, reactive oxygen species, inflammation

## Abstract

Increased oxidative stress plays a significant role in the etiology of bone diseases. Heightened levels of H_2_O_2_ disrupt bone homeostasis, leading to greater bone resorption than bone formation. Organochalcogen compounds could act as free radical trapping agents or glutathione peroxidase mimetics, reducing oxidative stress in inflammatory diseases. In this report, we synthesized and screened a library of organoselenium and organotellurium compounds for hydrogen peroxide scavenging activity, using macrophagic cell lines RAW264.7 and THP-1, as well as human mono- and poly-nuclear cells. These cells were stimulated to release H_2_O_2_, using phorbol 12-myristate 13-acetate, with and without organochalogens. Released H_2_O_2_ was then measured using a chemiluminescent assay over a period of 2 h. The screening identified an organoselenium compound which scavenged H_2_O_2_ more effectively than the vitamin E analog, Trolox. We also found that this organoselenium compound protected MC3T3 cells against H_2_O_2_-induced toxicity, whereas Trolox did not. The organoselenium compound exhibited no cytotoxicity to the cells and had no deleterious effects on cell proliferation, viability, or alkaline phosphatase activity. The rapidity of H_2_O_2_ scavenging and protection suggests that the mechanism of protection is due to the direct scavenging of extracellular H_2_O_2_. This compound is a promising modulators of inflammation and could potentially treat diseases involving high levels of oxidative stress.

## 1. Introduction

Oxidative stress plays a major role in the physiological decline of major body systems during aging [1,2], and in the pathology of chronic inflammatory diseases, including rheumatoid arthritis [3,4] and bone disorders, such as osteoporosis [5]. Immune cells such as mononuclear cells (MNC) and polymorphonuclear cells (PMNC), release multiple pro- and anti-inflammatory cytokines such as IL-1 and IL-6, as well as reactive oxygen species (ROS) such as hydrogen peroxide (H_2_O_2_), superoxide anions, and hydroxyl radicals, as part of the inflammatory response. ROS can lead to the destruction of foreign pathogens or act as intracellular signaling molecules, affecting proliferation, apoptosis, and differentiation. However, elevated levels and the prolonged exposure of tissue to H_2_O_2_ will lead to cell death and even organ failure [6]. 

Bone is a continuously remodeled tissue, resulting from the mineralizing action of osteoblasts and bone resorption by osteoclasts [7]. This equilibrium is governed by feedback mechanisms involving hormones, cytokines, and ROS [7,8,9,10]. ROS can potently stimulate osteoclastogenesis via RANKL/FoxO signaling [11,12]. H_2_O_2_ can negatively affect osteoblast differentiation, mineralization, and cause apoptosis [13,14,15]. Typically, ROS levels are tightly regulated by an enzymatic antioxidant system involving superoxide dismutase (SOD), glutathione peroxidase (GPx), and catalases. However, under inflammatory conditions, the bone equilibrium increasingly shifts to resorption, thus weakening the overall bone structure through micro-deterioration [16,17,18]. These inflammatory conditions present a significant monetary cost to the government and social institutions. Identifying and developing therapeutics that can attenuate oxidative stress within the body may ameliorate disease symptoms and improve patient care. 

Antioxidants such as Trolox (a water-soluble vitamin E analog) can be delivered to the body, supplementing the body’s natural oxidant defenses [19]. The incorporation of N-acetyl cysteine (NAC) into polymethyl methacrylate (PMMA) bone cements has reduced the cements’ cytotoxicity, reversed their negative effects on alkaline phosphatase activity, and upregulated osteoblastic genes [20,21]. The selenium (Se) which is incorporated into selenoproteins is an important part of the body’s antioxidant defense system. In the GPx enzymes, which catalyze the reduction of hydrogen peroxide to water, using glutathione (GSH) as the stoichiometric reductant, a selenocysteine residue is at the active site. A GPx-enzyme is also responsible for the reduction of lipid hydroperoxides to the corresponding alcohols [22]. Many drug development efforts [23] have been invested into finding simple organoselenium and organotellurium compounds that could mimic the action of the GPx-enzymes [24]. Small molecular selenium compounds (including Ebselen) have been studied to treat hearing loss [25], be cytoprotective against oxidative damage [26], and reduce mitochondrial damage in an acute stroke model [27]. Organotellurium compounds have been used as immunomodulatory, anti-inflammatory, and anti-apoptotic in Parkinson’s and diabetes models [28,29]. Previously, we synthesized organic compounds containing Se or Te which showed excellent antioxidative behavior [30,31,32,33,34]. These regenerable compounds greatly inhibited the rate of linoleic acid peroxidation in a two-phase peroxidation system, and showed GPx-like behavior in model systems.

While these chalcogen compounds performed admirably as antioxidants in pure chemical assays, their biological effects and applications have not been studied to a great extent [35]. In this paper, we screened several organoselenium and organotellurium compounds for their biological effects, in order to find potential therapies to treat inflammatory and bone diseases. Specifically, the goals of this study were to find compounds that could scavenge the H_2_O_2_ produced by immune cells, and assess whether they confer protection against H_2_O_2_-mediated toxicity, as well as any additional effects on the cell viability and bone forming activity (as indicated by alkaline phosphatase (ALP) activity) of a pre-osteoblastic cell line. To these ends, we identified a selenium-containing compound which met these criteria by scavenging released H_2_O_2_, protecting against H_2_O_2_-induced toxicity, without affecting ALP activity.

## 2. Materials and Methods

### 2.1. Organochalcogen Preparation

The structures of the organoselenium compounds **1**–**4** and organotellurium compounds **5**–**9** are shown in Table 1. The sources and synthesis steps of each compound are the same as previously described (see reference in Table 1). Each compound was dissolved in DMSO (Sigma-Aldrich, St. Louis, MO, USA) at a concentration of 10 mM and stored at −20 °C, prior to further dilution in cell medium.

### 2.2. Cell Preparation

All cell lines were purchased from American Type Cell Culture (ATCC, Manassas, VA, USA). Initial antioxidant screening was performed using two inflammatory cell lines: a mouse leukemic macrophage cell line (RAW264.7) and a human leukemic monocytic cell line (THP-1). Cell lines were maintained and expanded in T-75 flasks (VWR International, Radnor, PA, USA), in an incubator with a humidified atmosphere of 5% CO_2_ in air, at 37 °C. DMEM/F-12 medium (Thermo Scientific HyClone, Logan, UT, USA), supplemented with 10% fetal bovine serum (FBS) (Thermo Scientific Hyclone) and 1% penicillin/streptomycin (P/S, Thermo Scientific Hyclone), was used for feeding the RAW264.7 cells every other day, while the THP-1 cells were fed with RPMI 1640 medium (Thermo Scientific Hyclone), supplemented with 10%FBS/1%P/S. The RAW264.7 cells were used for experiments upon 80% confluence, while the THP-1 cells were maintained at a cell density between 1 × 10^5^ and 8 × 10^5^ cells/mL. Prior to plating, the RAW264.7 cells were detached by scraping in a single direction, using a cell scraper (MidSci, St. Louis, MO, USA).

To harvest human mononuclear cells (MNC) and polymorphonuclear cells (PMNC), primary cells from the blood buffy coat were obtained from the blood bank at Uppsala University Hospital. Ethical approval was not necessary, since the buffy coats were provided anonymously and can’t be traced to a single individual. This practice is in accordance with Swedish legislation (Act on Ethical Review of Research Involving Humans), section code 4§3p SFS 2003:460. The blood buffy coat was diluted in a 1:1 ratio with 1x phosphate-buffered saline (PBS, Fischer Scientific, Waltham, MA, USA), and mononuclear cells were isolated using Ficoll-Paque (Fischer Scientific), in addition to the density gradient centrifugation. Briefly, the diluted blood was placed on top of the Ficoll-Paque, and then centrifuged at 400 g for 30 min. After removing the plasma layer, the MNC layer was collected, washed with PBS, and centrifuged at 100 g for 15 min. This washing procedure was repeated for a total of three times. The total cell count was obtained using a hemocytometer and trypan blue exclusion method (Hyclone, Fischer Scientific). To isolate the PMNC, the blood was initially processed using Ficoll-Paque centrifugation, as described above. The blood pellet was then suspended in 3% dextran/0.9% saline (Sigma-Aldrich, St. Louis, MO, USA) for 20 min. The supernatant was collected and centrifuged at 250 g, for 10 min. To remove the erythrocytes, the pellet was washed with 0.2% saline solution for 20 s, and then an equal volume of 1.6% saline was added. The cells were spun at 250 g for 10 min and the pellet was resuspended in PBS. For counting, cell dilutions were prepared using a 1:1 dilution in 6% acetic acid (Sigma-Aldrich, Saint Louis, MO, USA), in order to distinguish PMNC from MNC.

### 2.3. Chemiluminescent Assay for Scavenging H_2_O_2_

The ability of the antioxidants to scavenge the H_2_O_2_ produced from activated inflammatory cells (both inflammatory cell lines and primary cells) was evaluated by means of a luminol-amplified chemiluminescence assay [38]. 

A 1% luminol stock solution was prepared by dissolving 50 mM of luminol (3-aminophthalhydrazide, Fisher Scientific) in 0.2 M NaOH. A cell suspension and luminol solution were prepared in a protein-free medium (4PBS:1DMEM/100 mM glucose). The luminol solution (500 µM) was prepared by adding luminol (from stock) and 0.2% horse radish peroxidase (HRP) (1 mg/mL) (Jackson Immuno Research, West Grove, PA, USA) to the medium. A total of 50 µL of the organochalcogen or Trolox (Sigma-Aldrich) solution was added to a 96-white well-plate (Perkin Almer, Waltham, MA, USA), together with 50 µL of a suspension of 2 × 10^5^ cells/well. A total of 100 µL of luminol solution, containing 1 µM phorbol-12-myristate-13-acetate (PMA, Sigma-Aldrich), was then added to the wells. As controls, cells in medium containing no organochalcogen were either added together with a luminol solution free of PMA (non-activated cells; negative control), or with a luminol solution containing PMA (activated cells; positive control). The luminescence was measured every 2 min by a microplate reader (Infinite M200, Tekan, Switzerland) set at 37 °C, using an integration time of 1500 ms and a settle time of 150 ms. To prevent exposure to light, the experimental procedure was performed in a dark room. Each experiment was performed in triplicate and repeated at least three times.

The relative amount of H_2_O_2_ that was generated over the course of the experiment was approximated by calculating the area under the luminescent curve. The area under the curve was determined using numerical integration (trapezoidal rule, Microsoft Excel, Redmond, WA, USA). Initially, the entire library of compounds was screened, and the antioxidants evaluated at a concentration of 10 µM that showed a higher scavenging effect, i.e., decreasing the H_2_O_2_ down to 30% of the control, were selected for additional tests. The scavenging effect of the selected antioxidants at lower concentrations (1, 5, and 10 µM) was evaluated. Each experiment was performed in triplicate and repeated at least three times.

### 2.4. Cytotoxicity Study

The RAW264.7and THP-1 cells were cultured with the selected antioxidants and the cell viability was determined after one and three days of culture. A total of 6500 RAW264.7cells per well were seeded in a 96-well plate (20,000 cells/cm^2^, VWR International). In the case of the THP-1 cells, the same cell density was seeded together with 20 nM PMA, to promote the differentiation of monocytes and hence, their attachment to the well. Cells were plated and fed using DMEM or RPMI-1640 medium, respectively. At 3 h after cell seeding, the medium was exchanged with medium containing the selected organochalcogen. The concentrations of the organochalcogen compounds used were 1, 5, and 10 µM. At each time point, the wells were washed with PBS and incubated with 200 µL 5% Alamar Blue (Invitrogen, Carlsbad, CA, USA) in DMEM medium for 1 h at 37 °C, and then the fluorescence was monitored by a microplate reader Tecan (Infinite M200, Tecan, Männedorf, Switzerland), at 560 nm excitation and 590 nm emission. The fluorescence signal was converted to cell numbers using a calibration curve. To assess the relative viability of MC3T3-E1 (ATCC) after exposure to the antioxidants, cells were seeded in 96-well plates (10,000 cells/cm^2^), and were fed every other day with Mem-alpha (Thermo-Fischer, Waltham, MA, USA), and different concentrations of organochalcogens and Trolox (Sigma-Aldrich). On days 3, 7, 10, and 14, the cells were incubated with AlamarBlue. Experiments were repeated at least three times, with *n* = 6 per sample per experiment.

### 2.5. H_2_O_2_ Toxicity Assay

To determine the concentration of H_2_O_2_ that is toxic to a preosteoblastic cell line, the MC3T3-E1 cells were seeded (10,000 cells/cm^2^) in 96-well plates using MEM alpha medium. At 24 h after plating, the MC3T3-E1 cells were treated with different concentrations of H_2_O_2_ (Sigma-Aldrich, 0, 1, 10, 100, 300, and 600 μM) for 3 h, and then washed with fresh medium. At 24 h and 72 h after the wash, cells were incubated with AlamarBlue (1:20 dilutions) in cell medium for 1.5 h, and then the fluorescence was measured using a plate reader. To assess whether the organochalcogen could protect against the effects of H_2_O_2_, cells were plated at the same densities and the organochalcogen compounds were co-treated at the same time as H_2_O_2_. Experiments were repeated at least three times, with *n* = 6 per sample per experiment.

### 2.6. Alkaline Phosphatase (ALP) Activity

The MC3T3-E1 cells were plated in 96-well plates (10,000 cells/cm^2^), over a period of 14 days. Cells were fed with Mem alpha, supplemented with ascorbic acid (50 μg/mL, Sigma-Aldrich) and β-glycerolphosphate (10 mM, Sigma-Aldrich). On days 3, 7, 10, and 14, after each Alamar blue assay, each plate was washed once with PBS, covered with 200 μL of lysis buffer (20 mM Tris, 1 mM MgCl_2_, 0.1 mM ZnCl_2_ and 0.1% Triton X-100, all from Sigma-Aldrich), and then stored at −20 °C. Each plate underwent three freeze-thaw cycles, prior to the measuring of the total protein content and ALP activity. The total protein content was quantified using a microBCA kit (Thermo-Fischer), according to manufacturer’s instructions. The ALP activity was measured by incubation with alkaline phosphatase yellow (Sigma-Aldrich) for 20 min in the dark at 37 °C, stopping the reaction with 0.1 mM NaOH (Sigma-Aldrich), and the absorbance readings were taken at 405 nm. Both the total protein content and the ALP activity were determined using a standard curve, and the ALP activity was normalized by the protein content and duration of assay incubation. Experiments were repeated at least three times, with *n* = 6 per sample per experiment.

### 2.7. Statistical Method

Statistical analysis was performed using PSPP software (GNU Project, Free Software Foundation). Significance was determined by *p* < 0.05. To compare the results of different groups, we performed one way ANOVAs and then a Scheffe posthoc test. 

## 3. Results

### 3.1. C1 and C7 Significantly Reduced H*_2_*O*_2_* Generated from Immune Cell Lines

**Figure 1:**
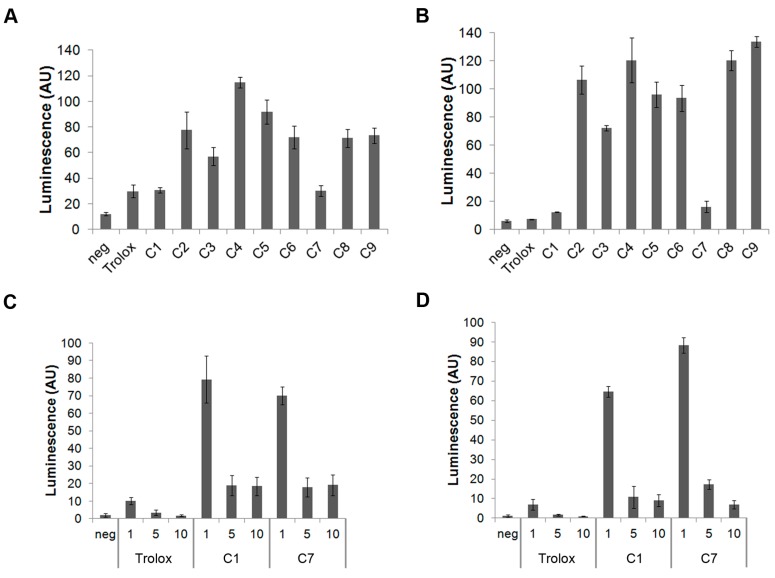
Luminescence detected when cells were: untreated (negative), treated with PMA (1 μM) alone, or with an antioxidant (10 μM) in RAW264.7 cells (**A**); or THP-1 cells (**B**). The luminescence produced by RAW264.7 cells (**C**) or THP-1 cells (**D**) when exposed to Trolox, C1, or C7 at 1, 5, or 10 µM. In both figures the values are presented normalized to the positive control, and expressed as %. Values expressed as mean ± standard deviation.

Initial antioxidant screening using activated RAW264.7 and THP-1 cells revealed that C1 and C7 significantly decreased the H_2_O_2_-induced luminescence, compared to the positive control (cells activated with 1uM PMA) (Figure 1A,B), and were comparable to Trolox. Specifically, for the RAW264.7 cells, C1 and C7 were able to reduce H_2_O_2_ to 30% of what was generated in the positive controls. These two compounds showed a greater reduction in THP-1 cells compared to the RAW264.7 cells, with only 16% of H_2_O_2_ remaining unquenched in the THP-1 cells. The remaining compounds (C2–6, C8, C9) were equally efficient quenchers of H_2_O_2_, in each of the cell types (Figure 1A,B). Since C1 and C7 were significantly more effective, the remaining compounds were not retained in subsequent tests. The dose response of H_2_O_2_ reduction after Trolox, C1, or C7 exposure, in the Raw264.7 and THP-1 cells, are shown in Figure 1C,D, respectively. In both the RAW264.7 and THP-1 cells, it was observed that an optimal quenching occurred at, and above, 5 μM, while 1 µM did not significantly lower H_2_O_2_ levels. C1, C7, and Trolox exhibited comparable scavenging efficiency in both cell types (Figure 1C,D).

**Figure 2:**
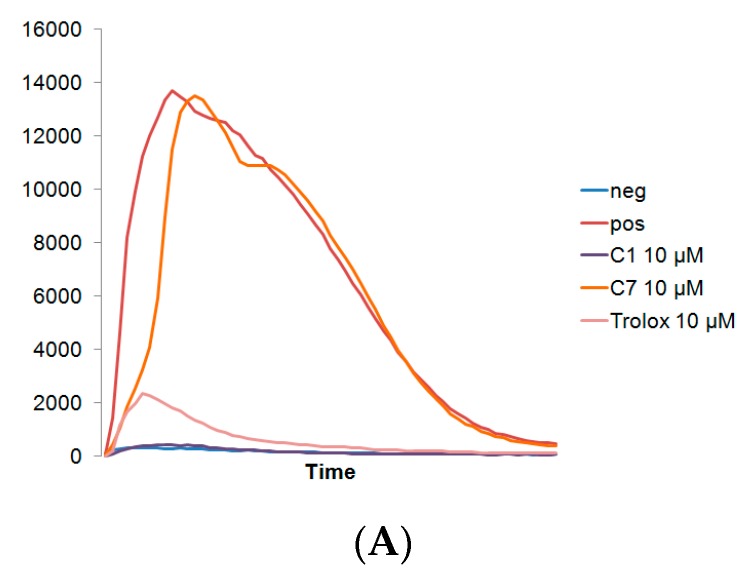

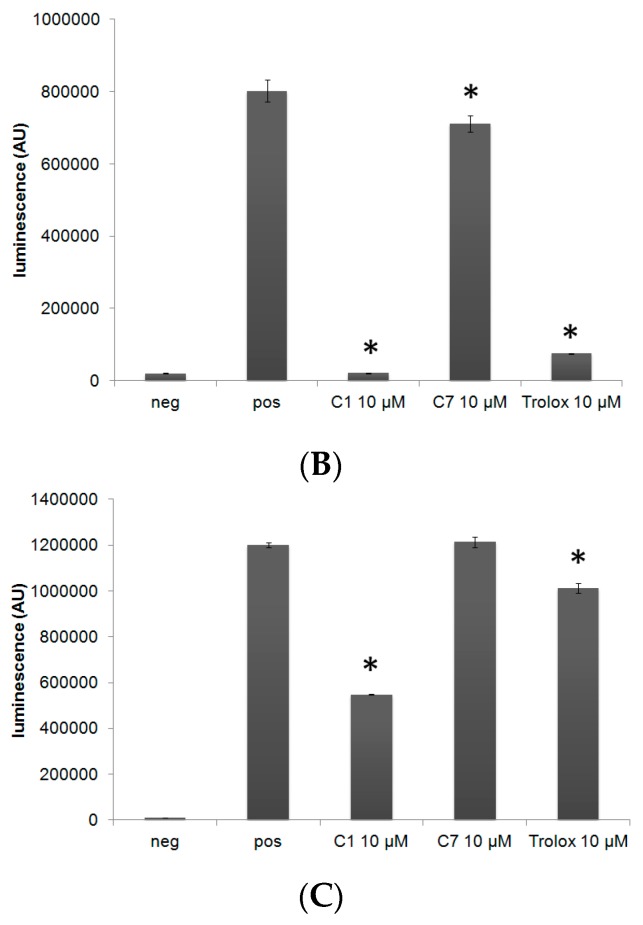
(**A**) Representative curve of H_2_O_2_ generation as detected by chemiluminesence over 2 h by MNC. Reduction of H_2_O_2_ by C1, C7, and Trolox in (**B**) MNC and (**C**) PMNC, * indicates *p* < 0.05 reduction compared to positive control. Values expressed as mean ± standard deviation.

Human MNC and PMNC typically produce more ROS than carcinoma cell lines. C1 was able to significantly reduce the H_2_O_2_ generated from both MNCs and PMNCs, at all tested concentrations, and, in fact, was more effective than Trolox. While C7 was effective in reducing the H_2_O_2_ derived from MNCs, it was not able to significantly reduce the H_2_O_2_ from PMNCs at the tested concentrations (Figure 2).

### 3.2. C1 and C7 Did Not Significantly Affect Cell Viability

**Figure 3:**
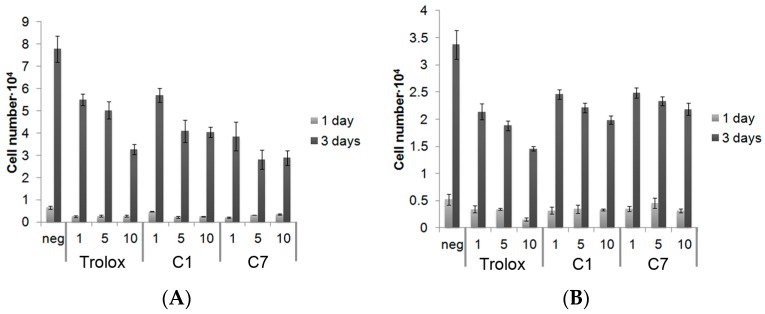
Viability of (**A**) RAW264.7 cells and (**B**) THP-1 cells after exposure to Trolox, C1, and C7 at different concentrations (1, 5, and 10 µM) (normalized to positive control). Values expressed as mean ± standard deviation.

Initial toxicity screening of the organochalcogen was conducted using AlamarBlue assay on the RAW264.7 and THP-1 cells. The cell number after culturing the RAW264.7 cells and THP-1 with antioxidants at different time points, is shown in Figure 3. No significant differences in cell numbers between the different treatment groups was observed on day one. By day three, all groups had increased their cell numbers, compared to day one. Compound C7 slightly reduced the proliferation rate of RAW264.7, while Trolox slowed cell growth more than either of the compounds in THP-1 cells.

### 3.3. C1 Protects MC3T3-E1 Cells against H*_2_*O*_2_* Mediated Toxicity

**Figure 4:**
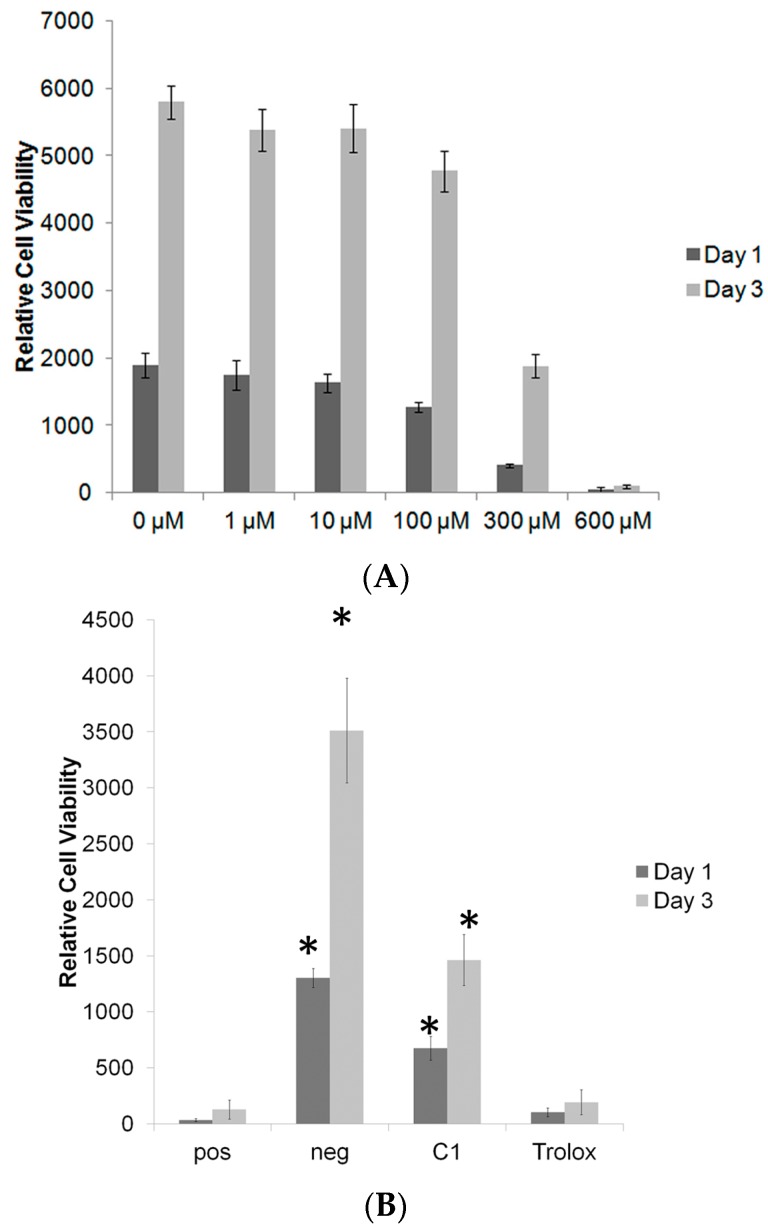
(**A**) Toxicity of different concentration of H_2_O_2_ on MC3T3-E1 (**B**) Effect on MC3T3-E1 protection against H_2_O_2_ (pos control) by C1 and Trolox (both at 25 μM), * indicates *p* < 0.05 compared to positive control. Data expressed as mean ± standard deviation.

Antioxidants that do not negatively affect cell viability, may still impair cell differentiation. Thus, we examined the effects of each antioxidant on bone cell viability and differentiation, using MC3T3 pre-osteoblasts. To assess whether C1 can protect MC3T3s against H_2_O_2_-induced cell death, we treated the cells with hydrogen peroxide and C1 or Trolox. A dose response study with H_2_O_2_ revealed that 300 μM or higher of H_2_O_2_ was necessary, in order to induce significant cell toxicity. When cells were treated with toxic levels of H_2_O_2_ and slightly higher levels of antioxidant (25 μM, Figure 4), C1, but not Trolox, was able to partially protect MC3T3-E1 against H_2_O_2_ (300 μM) mediated toxicity.

### 3.4. Exogenous Application of Antioxidants Does Not Affect Endogenous Proliferation or ALP Activity of MC3T3s

**Figure 5:**
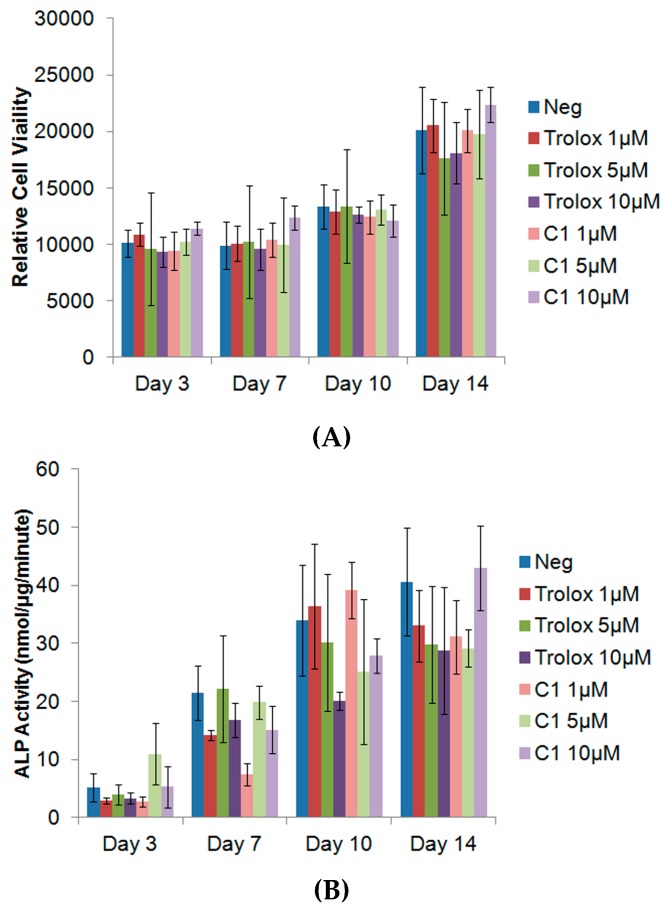
Cell Viability of MC3T3-E1 after exposure to antioxidants C1 and Trolox (**A**) and ALP activity after exposure to antioxidants C1 and Trolox (**B**). Data expressed as mean ± standard deviation.

To determine whether C1 would affect the normal proliferation and bone mineral production of MC3T3-E1s, cells were exposed to different concentrations of antioxidants, and the proliferation, along with the ALP activity, was measured over the course of 14 days. As can be seen from Figure 5, no significant adverse effect of the antioxidants on the relative cell viability was observed, at any time point. There was also no difference in the proliferative rate between any of the experimental groups and the control. ALP activity is a widely used indicator of new bone mineralization. A decrease in ALP activity would suggest that the antioxidant may impair new bone mineral formation. However, no significant differences were observed in the ALP activity between any of the groups. Given that there was no difference in the ALP activity, additional assays such as alizarin red, which are used to assess mineralization, were not warranted. These results indicate that the antiodixants C1 and C7, do not negatively affect the proliferative or ALP activity of MC3T3-E1 osteoblasts. 

## 4. Discussion

A library of organoselenium and organotellurium antioxidants were screened for their ability to scavenge the H_2_O_2_ released from activated leukocytes, and their effects on the cellular toxicity and ALP activity on preosteoblastic MC3T3-E1 cell lines. The inhibition of peroxidation and GPx-like activity of these compounds had been previously assessed [32], but without an extensive study of how these compounds behave in biological systems. In addition to chronic inflammatory conditions, excessive oxidative stress can also occur after the implantation of a biomedical device, such as bone cement. Depending on the material, cements, as well as their degradation products, can generate free radicals, stimulating an exaggerated inflammatory response and oxidative stress that can lead to severe cell toxicity and suppress osteoconductivity; this can be restored by enhancing the cellular proliferation and differentiation of osteoblasts [20,21,39]. Major inflammatory sources of extracellular ROS include leukocytes, such as PMNC and MNC, that are recruited to the site of implantation. 

The organoselenium C1 and the organotellurium compound C7 quickly scavenged the H_2_O_2_ generated from the PMA-activated cell lines, whereas only C1 performed well against primary human leukocytes and protected osteoblast precursors against hydrogen peroxide. These antioxidative effects may be due to the ability of these compounds to act as radical trapping agents [25,26], and the capacity of the organotellurium compound to mimic the action of the GPx-enzymes [32]. A previous study has shown that Trolox is likely to exert protection against intracellular H_2_O_2_ by intracellular scavenging [40]. In our experimental model, the cells were exposed to the antioxidants and peroxide at the same time, which seems to indicate that it is the extracellular scavenging of peroxide which confers the protection seen with C1. Pre-incubation may be important for providing Trolox with enough time to enter the cells, thus protecting them. 

The mechanism underlying how different types of antioxidants affect osteogenesis and osteoblast activity is more complex. Oxidative stress due to intracellular H_2_O_2_ has long been hypothesized to have a major influence on bone density [41]. NAC has been able to protect osteoblasts from H_2_O_2_-induced oxidative stress [42], and increase the ALP activity of rat bone marrow stromal cells by enhancing osteoblastic differentiation [43]. This latter work also demonstrated that NAC exposure led to a higher gene expression of BMP-2, Runx2, collagen I, osteopontin, and osteocalcin. However, ROS is also a critical signalling factor for multiple cell processes and an inevitable by-product of the mitochondrial electron transport chain [44]. Tight control of the glutathione redox system was shown to be particularly important in osteogenic activity [45]. ROS generated from BMP-2 is required to stimulate 2T3 pre-osteoblasts cells’ differentiation, and the application of NAC may decrease ALP expression [46]. Another antioxidant, α-tocopherol, a vitamin E analog, was actually shown to decrease the ALP expression of osteoblasts isolated from rat calvariae [47]. 

Other antioxidants, such as α-tocotrienol, suppressed RANKL expression and osteoclastogenesis [48]. Studies using a vitamin E deficient mouse model (Ttpa-/-) found increased bone mass due to reduced osteoclast fusion [49]. In this study, α-tocopherol (and not vitamin E congeners), was found to affect osteoclast formation during the late maturation phase, suggesting that it is not the antioxidant activity which affects osteoclastogenesis. Although H_2_O_2_ is probably the least reactive of the various ROS, it does form in large amounts, and can cause considerable damage [50]. H_2_O_2_ suppresses ostebolastic differentiation, ALP expression, type 1 collagen, and colony-forming unit-osteoprogenitor formation, via ERK- and ERK-dependent NF-κB activation [51]. Oxidative stress activation of NF-κB and PKCβ/p66SHC signalling also stimulates ostebolastic apoptosis [11]. Similarly, H_2_O_2_ may antagonize Wnt signalling and attenuate osteoblastogenesis with age [52]. In this preliminary screening, bone cell proliferation and mineralization do not appear to be impaired by the most potent antioxidants, C1 and C7. Both compounds performed comparably to, or better than, one of the most potent, cytocompatible antioxidants, Trolox. Whether, and how, these organochalcogen compounds affect gene expression during osteoblastic differentiation and osteoclastogenesis, will be the subject of future studies. 

## 5. Conclusions

In summary, we have identified an organoselenium compound which could efficiently quench the levels of H_2_O_2_ generated from both cell lines and primary human blood cells, at comparable or lower levels than Trolox. This compound also protected MC3T3-E1 against H_2_O_2_-induced toxicity, without affecting cell viability or ALP activity, and could potentially be incorporated into an implanted biomaterial to reduce the level of inflammation, or be used for developing therapies for diseases involving high levels of oxidative stress.

## Figures and Tables

**Table 1:** Structure and origin of tested compounds.

Compound	Structures	Reference
**C1**	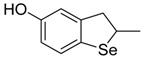	[31]
**C2**	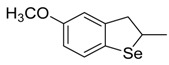	[31]
**C3**	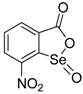	[36]
**C4**	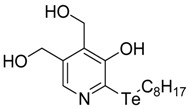	[37]
**C5**	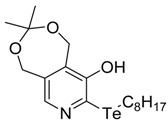	[37]
**C6**	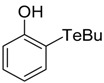	[32]
**C7**	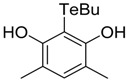	[32]
**C8**	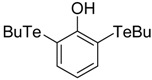	[32]
**C9**	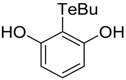	[32]
